# Tetramethylpyrazine nitrone, a multifunctional neuroprotective agent for ischemic stroke therapy

**DOI:** 10.1038/srep37148

**Published:** 2016-11-14

**Authors:** Zaijun Zhang, Gaoxiao Zhang, Yewei Sun, Samuel S. W. Szeto, Henry C. H. Law, Quan Quan, Guohui Li, Pei Yu, Eiketsu Sho, Michael K. W. Siu, Simon M. Y. Lee, Ivan K. Chu, Yuqiang Wang

**Affiliations:** 1 Institute of New Drug Research and Guangzhou Key Laboratory of Innovative Chemical Drug Research in Cardio-cerebrovascular Diseases, Jinan University College of Pharmacy, Guangzhou, 510632, China; 2 Department of Chemistry, the University of Hong Kong, Hong Kong, China; 3 Kunming Biomed International and National Engineering Research Center of Biomedicine and Animal Science, Kunming, 650500, China; 4 Department of Chemistry and Biochemistry, University of Windsor, Windsor, Ontario, Canada; 5 State Key Laboratory of Quality Research in Chinese Medicine, Institute of Chinese Medical Sciences, University of Macau, Macau, China

## Abstract

TBN, a novel tetramethylpyrazine derivative armed with a powerful free radical-scavenging nitrone moiety, has been reported to reduce cerebral infarction in rats through multi-functional mechanisms of action. Here we study the therapeutic effects of TBN on non-human primate model of stroke. Thirty male *Cynomolgus* macaques were subjected to stroke with 4 hours ischemia and then reperfusion. TBN were injected intravenously at 3 or 6 hours after the onset of ischemia. Cerebral infarction was examined by magnetic resonance imaging at 1 and 4 weeks post ischemia. Neurological severity scores were evaluated during 4 weeks observation. At the end of experiment, protein markers associated with the stroke injury and TBN treatment were screened by quantitative proteomics. We found that TBN readily penetrated the blood brain barrier and reached effective therapeutic concentration after intravenous administration. It significantly reduced brain infarction and modestly preserved the neurological function of stroke-affected arm. TBN suppressed over-expression of neuroinflammatory marker vimentin and decreased the numbers of GFAP-positive cells, while reversed down-regulation of myelination-associated protein 2′, 3′-cyclic-nucleotide 3′-phosphodiesterase and increased the numbers of NeuN-positive cells in the ipsilateral peri-infarct area. TBN may serve as a promising new clinical candidate for the treatment of ischemic stroke.

Stroke is one of the most devastating diseases affecting the health and life of human beings. Despite the remarkable progresses achieved in the last two decades in understanding the pathophysiology of stroke, tissue-type plasminogen activator (tPA) remains the sole therapeutics approved by the US FDA for the treatment of acute ischemic stroke. However, tPA has a narrow therapeutic window of 3–4.5 h within the occurrence of a stroke, limiting its clinical use. Besides, tPA has no neuroprotective effect. Although many pharmacological agents for stroke have shown considerable promise in rodent models, none have successfully been translated for use in humans^1^. Nevertheless, finding an effective neuroprotective agent remains a strongly sought after goal^2^,^3^.

One of the most extensively studied classes of neuroprotective agents is free radical scavengers, as free radical over-production is a major contributor to brain injury after stroke. NXY-059, a free radical-trapping nitrone, showed positive results when evaluated in various animal stroke models but failed its second phase III clinical trial^4^,^5^. The cause of this failure is multifactorial, including poorly designed preclinical and clinical studies^6^ and its poor ability in penetrating the blood-brain barrier (BBB)^7^,^8^. Edaravone, also a free radical scavenger, has been approved for treatment of patients with ischemic stroke in Japan^9^ and China. However, it is not approved for use in Western countries due to limited efficacy and side effects, such as acute renal failure, liver dysfunction and acute allergic reactions^10^.

Tetramethylpyrazine (TMP), a main active ingredient of *Ligusticum wallichii* Franchat (*Chuanxiong*), has been widely used as a treatment of ischemic stroke for a long time in China^11^. However, until now, there has not ever been a methodologically sound clinical trial demonstrating its benefit. Although the exact mechanisms of action of TMP have not been completely understood, a variety of mechanisms may be attributed to its beneficial effects in stroke patients, including inhibiting platelet aggregation, lysing blood clots, blocking intracellular calcium entry and scavenging free radicals^12^. Nitrone is originally developed as free radical-trapping agents in free radical chemistry, potently scavenges free radicals and protects biological systems from oxidative stress^8^. Nitrones have been tested as therapeutic agents for neural and systemic dysfunctions including atherosclerosis, septicemia, stroke, and Alzheimer’s disease^13^,^14^. Taking advantage of the beneficial effects of both TMP and nitrone, therefore, we designed and synthesized (2-[[(1,1-dimethylethyl)oxidoimino]-methyl]-3,5,6-trimethylpyrazine, TBN), a TMP derivative armed with a powerful free radical-scavenging nitrone moiety. TBN was desired to possess the pharmacological properties of both TMP and nitrone.

We previously demonstrated that TBN protected rats from ischemic stroke damage^15^,^16^. A multi-functional mechanisms possibly contributed to TBN’s beneficial effects in stroke models, including directly scavenging various free radicals, blockade of calcium entry and inhibition of platelet aggregation^16^. To bridge the biological gap between the rodent animal experiments and clinical trials, in the present study, we investigated the therapeutic efficacy of TBN in non-human primate (NHP) model of ischemic stroke, as well as its pharmacokinetics, fulfilling the requirements for initiating investigational new drug application.

## Results

### TBN reduces cerebral infarction and improves neurological functions in stroke monkeys

**Figure 1:**
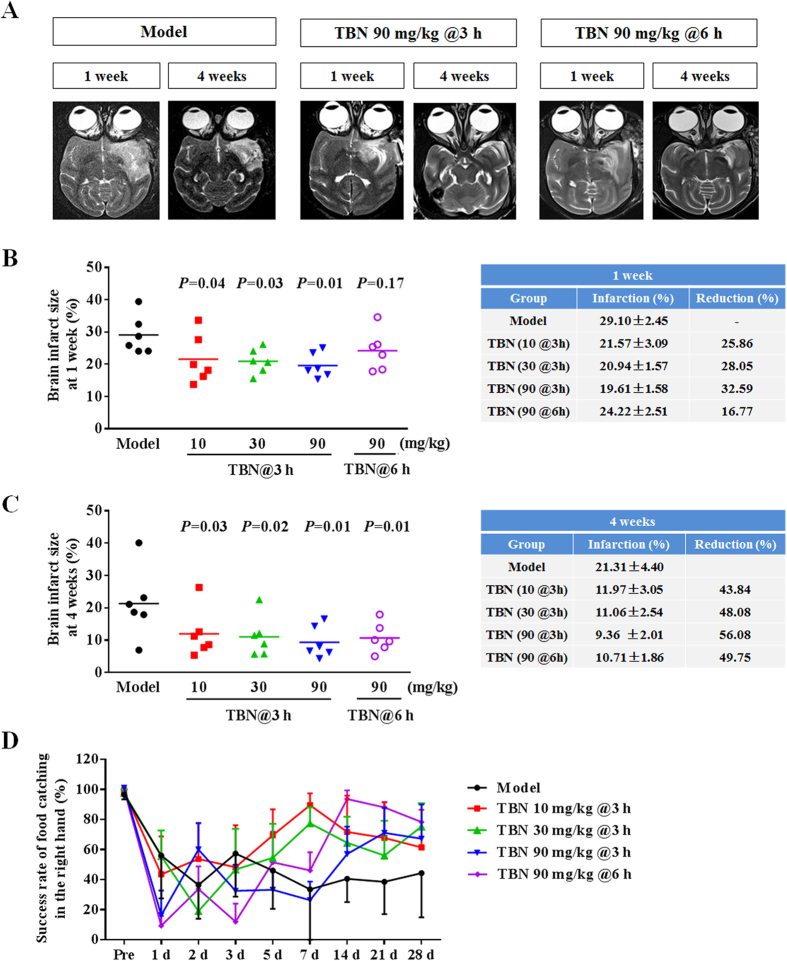
TBN attenuates brain infarct volume and improves neurological function in monkeys subjected to *t*-MCAo. (**A**) Representative images of *T*_*2*_-weighted MRI scan. (**B**,**C**) Brain infarct size of ipsilateral hemisphere. Data were expressed as dot plots with means in the left panel and mean ± s.e.m. in the right panel (n = 6 for each group). (**D**) Success rate of food catching of the contralateral, stroke-affected hand. Data were expressed as mean ± s.e.m. (n = 6 for each group). ‘@3 h’ and ‘@6 h’ mean TBN treatment initiating at 3 h and 6 h post-ischemia.

We evaluated TBN in *M. fascicularis* (cynomolgus macaque) subjected to *t*-MCAo. At one week post-surgery, TBN treatment administered at 3 h post-ischemia caused a reduction in overall infarct size in a dose-dependent manner as measured by *T*_*2*_-weighted MRI scans (Fig. 1A,B). Since only a small percentage (~3%) of stroke patients receive therapeutic intervention within 3 h after onset of ischemic stroke^17^, it was important to establish whether TBN is effective even under delayed treatment scenarios. TBN was found to be effective even when administered 6 h post-ischemia, indicating the potential for an expanded therapeutic window (Fig. 1A,B). At four weeks, similar dose-dependent trends were observed, with all treatments resulting in significant reductions in infarct size (Fig. 1A,C).

Functional neurological assessments were performed throughout the 28-day observation period, which ascertained that TBN treatment ameliorated the long-term neurological impairment produced by *t*-MCAo. Monkeys treated with TBN trended to have lower neurological deficit scores at 1 and 4 weeks post-ischemia (Table 1). Scores included assessments of consciousness, sensory and motor system function, and skeletal muscle coordination. Impressively, at 4 weeks post-ischemia, both the 30 and 90 mg kg^−1^ TBN treatment regiments produced a ~50% recovery of arm function, as demonstrated by the restored ability of the contralateral, stroke-affected arm to acquire food (Fig. 1D and see Supplementary Movie 1 and 2).

### TBN readily penetrates the blood-brain barrier and reaches therapeutic concentration

**Figure 2:**
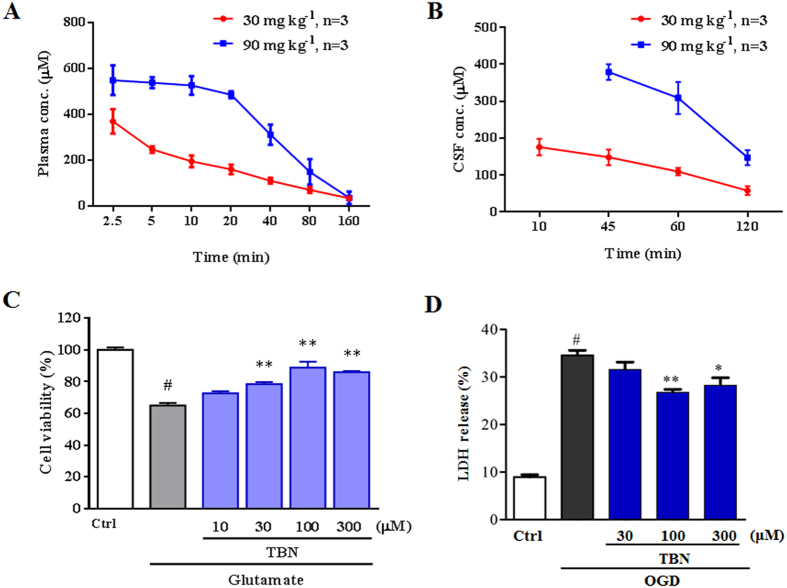
TBN readily penetrates the blood-brain barrier and reaches neuroprotective concentrations. (**A**,**B**) Blood and brain cerebrospinal fluid (CSF) concentrations *vs.* time profile after drug administration. (**C**) TBN protects primary cortical neurons against glutamate-induced excitotoxicity. ^#^*P *< 0.05 versus control (Ctrl); ^**^P < 0.01 versus glutamate treatment alone. Data were from three independent experiments. (**D**) TBN attenuates OGD-induced LDH release in primary cortical neurons. ^#^*P *< 0.05 versus control (Ctrl); ^*^P < 0.05 and ^**^P < 0.01 versus OGD alone. Data were from three independent experiments.

One reason that NXY-059 failed in clinical trials was its poor penetration of the BBB, with only 1% penetration in rats^8^. To investigate its ability to penetrate the BBB, TBN’s concentration in the monkey plasma and cerebrospinal fluid were examined. After intravenous (*i.v.*) administration of 30 and 90 mg kg^−1^ in monkeys, the plasma C_max_ of TBN reached 370 and 549 μM, respectively (Fig. 2A). In the CSF, the concentrations for the 30 and 90 mg kg^−1^ TBN treatment regiments were 176 μM at 15 min and 379 μM at 45 min, respectively (Fig. 2B). At 2 h after administration, TBN was still present at concentrations of 58 and 148 μM, respectively. TBN concentrations achieved *in vivo* were at similar levels to those (30–300 μM) that protected cortical neurons against glutamate-induced excitotoxicity effectively (Fig. 2C), and attenuated oxygen and glucose deprivation (OGD)-induced release of lactate dehydrogenase (LDH) in cortical neurons (Fig. 2D).

### Characterization of molecular events involved in neuroprotection by iTRAQ-based quantitative proteomic analyses

**Figure 3:**
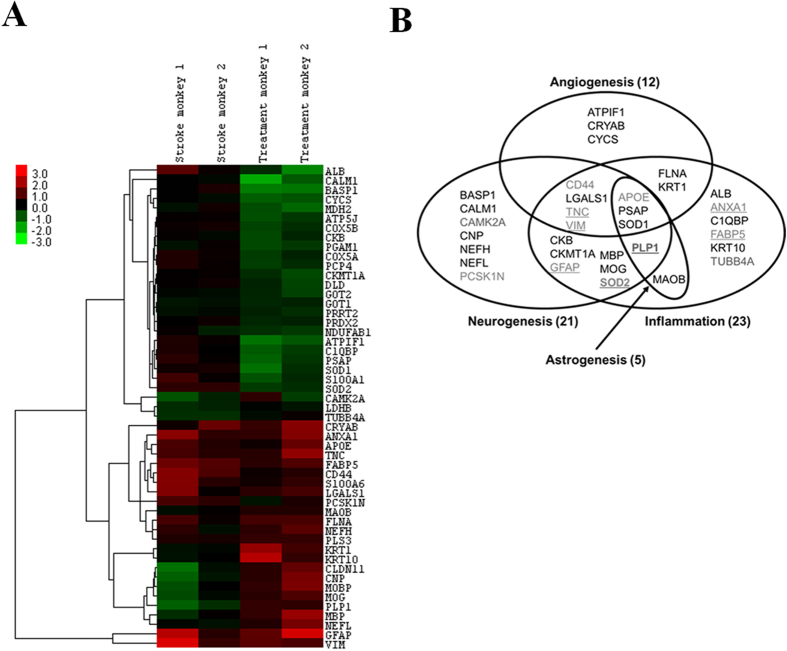
Dysregulated proteins in quantitative proteomic analyses functionally annotate to cellular processes associated with the chronic phase of stroke. (**A**) Hierarchical cluster analysis of dysregulated proteins for the stroke peri-infarct tissue versus the contralateral normal tissue and the TBN-treated peri-infarct tissue versus the contralateral normal tissue. (**B**) Dysregulated proteins functionally annotate to cellular processes of angiogenesis, neurogenesis, astrogenesis and inflammation.

To gain insight into the molecular mechanisms underlying TBN’s neuroprotection *in vivo*, the cerebral tissues of the untreated and 30 mg kg^−1^ TBN-treated monkeys were subjected to iTRAQ-based quantitative proteomic analyses (see Supplementary Fig. S1). A total of 2935 non-redundant proteins were confidently identified (local FDR <5%) (see Supplementary Dataset 1), with the differential expression profiles of 1497 quantified proteins compared in the model peri-infarct tissue versus the contralateral normal tissue and the TBN-treated peri-infarct tissue versus the contralateral normal tissue (see Supplementary Dataset 2). Seven and 37 proteins were observed to be dysregulated exclusively in the untreated and TBN-treated cerebral tissues, respectively, with 2 proteins showing reciprocal expression and 5 proteins being upregulated in both sample groups (see Supplementary Table 1 and Supplementary Dataset 3). Thirty-three of these 51 dysregulated proteins were functionally annotated to cellular processes associated with the chronic phase of stroke, including angiogenesis, neurogenesis, astrocytosis and inflammation^18^,^19^,^20^,^21^,^22^ (Fig. 3). These results revealed the major cellular processes associated with the observed proteomic changes. Interestingly, 8 of the 18 dysregulated proteins that were not annotated to the aforementioned processes are associated with various aspects of cellular energy metabolism and mitochondria. All were downregulated after TBN treatment (see Supplementary Table 2).

**Figure 4:**
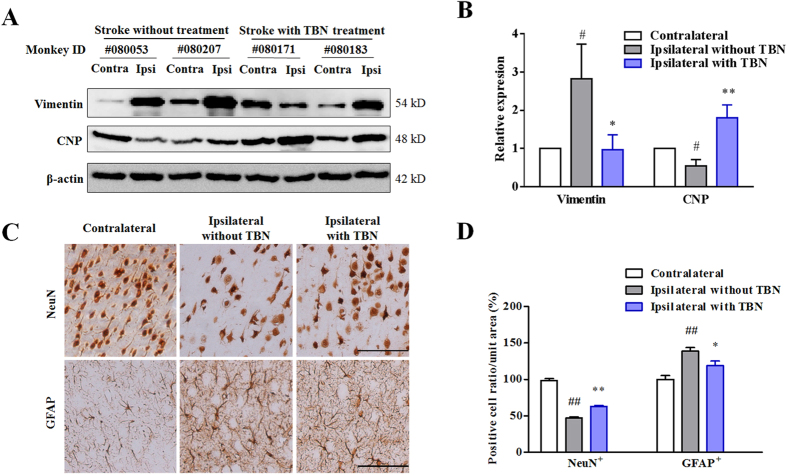
Molecular and cellular events involved in the protection of TBN against stroke injury. (**A**) Representative immunoblots of monkey brain tissue. (**B**) Densitometric quantification of the immunoblots as shown in Supplementary Fig. S1. ^#^*P *< 0.05 verses contralateral (Cont); ^*^P < 0.05 and ^**^P < 0.01 verses ipsilateral (Ipsi) without TBN treatment. (**C**) Representative immunohistochemistry images of cortical sections. Scale bars, 50 μm. (**D**) Quantification of the number of NeuN and GFAP positive cells. Data were expressed as percentage of sham group (n = 5 for Contralateral; n = 4 Ipsilateral without TBN and n = 6 for Ipsilateral with TBN). ^##^*P *< 0.01 verses sham group; ^*^P < 0.05 and ^**^P < 0.01 verses model group.

Two of the significantly dysregulated proteins, namely vimentin and 2′,3′-cyclic-nucleotide 3′-phosphodiesterase (CNP) were chosen for further validation using Western blotting, as changes of both vimentin and CNP have been previously reported in rodent models of ischemic stroke^23^,^24^,^25^. Vimentin expression was markedly up-regulated in the ipsilateral (Ipsi) region of the brain compared to that of the contralateral part. TBN treatment notably suppressed the up-regulation of vimentin in the ipsilateral region (Fig. 4A,B and see Supplementary Fig. S2). Vimentin is a component of the intermediate filament system and a prominent marker of microglia and reactive astrocytes^23^. Up-regulation of vimentin demonstrates that microglia and reactive astrocytes are activated and/or are proliferating in response to the presence of injured neural tissues (Fig. 4A,B and see Supplementary Fig. S2). Microglia and astrocytes activation and proliferation were further confirmed by the increased presence of GFAP^+^ cells by immunohistochemistry (Fig. 4C,D). Unlike vimentin expresson, the expression level of CNP was dramatically decreased in the stroke peri-infarct tissue in comparison to that in the contralateral normal tissue (Fig. 4A,B and see Supplementary Fig. S2). CNP is a highly expressed enzyme in the myelin sheath and is believed to play an essential role in axon myelination^24^ (Fig. 4A,B and see Supplementary Fig. S2). The decreased CNP expression implies neuronal damage and demyelination in stroke^24^,^26^. TBN treatment caused a reversal in the expression of CNP (Fig. 4A,B and see Supplementary Fig. S2), suggesting possible neuronal repair or regeneration by TBN. In accordance with this finding, TBN treatment notably increased the number of NeuN^+^ neurons with respect to the stroke model group (Fig. 4D,E).

## Discussion

The high-order brain of NHP is anatomically and functionally similar to that of humans and is amenable to quantitative assessment of the ischemic damage *via* MRI^17^. In addition, comprehensive functional evaluations using standardized tests are available due to the monkey’s extensive behavioral repertoire^17^. Researchers hope that monkey data will advance the field of stroke therapy with neuroprotective agents. NA-1(also termed Tat-NR2B9c), a 20-mer peptide functioned as a PSD-95 inhibitor, has been assessed in stroke model macaques monkey and now advances into Phase 3 clinical trial^2^. In reporting NA-1, Cook and his colleagues found that monkeys treated with NA-1 exhibited a significant reduction in infarct volume compared with placebo (38.7% reduction by *T*2-weighted MRI scans at 30-day post-stroke), NA-1 was effective even when administrated up to 3 h after the onset of stroke^2^. In our present study, TBN effectively reduced brain infarction by 43.84–56.08% reduction measured by *T*2-weighted MRI scans at 28-day post-stroke. More importantly, TBN was still effective administrated up to 6 h after the onset of stroke, suggesting that more patients can be treated. In addition to significantly reduced brain infarction, TBN treatment also trended to recover the food-catching function of stroke-affected arm. Severe contralateral hemiparesis is a long-lasting and typical clinical presentation after ischemic stroke, with functional recovery of the hemiplegic limb considered as a hallmark indicator for evaluation of the efficacy and prognosis of stroke therapeutics^27^. Our result suggests that TBN possibly improves the major clinical manifestations of the stroke subjects.

One of the principle features of an effective stroke neuroprotective agent is its ability to penetrate the BBB to reach the site of action. NXY-059 has two negatively charged sodium sulfonate moieties, and it is well known that negatively charged compounds hardly penetrating BBB^6^,^8^. To effectively deliver the free radical-scavenging nitrone moiety into brain tissues, TBN was designed using TMP as a carrier. TMP’s superior ability to enter the brain was implied as early as 500 years ago in the Compendium of Materia Medica (Bencao Gangmu). Using a microdialysis method, Tsai *et al.* demonstrated that TMP effectively penetrated the BBB, yielding a progressively higher brain/blood ratio from 10 to 120 min following intravenous administration^28^. In our present study, pharmacokinetic analysis in monkeys demonstrated that TBN readily penetrated the BBB and reached therapeutic concentrations in targeted cerebral tissues. This feature of TBN is pivotally important accounting for its high therapeutic efficacy.

In addition to taking advantage of TMP’s superior capability to penetrating BBB to deliver the nitrone into brain, we also take TMP’s another property, namely its ability to inhibit Ca^2+^ influx into cells. Thus, TBN can effectively enter the injured brain tissues to both inhibit Ca^2+^ influx and scavenge free radicals, two major causes of ischemic neuronal damage^29^. Previously, we have demonstrated that a multi-functional mechanism, including direct scavenging free radicals, blockade of intracellular Ca^2+^ entry, as well as inhibition of platelet aggregation, possibly contributed to TBN’s neuroprotective effects in cellular and in rodent stroke models^16^.

Tetramethylpyrazine (TMP) has been widely used as a treatment of ischemic stroke for a long time in China. A Chinese meta-analysis evaluated the safety of TMP injection plus conventional medicine for acute cerebral ischemic stroke. Compared to conventional medicine treatment alone, TMP injection (80–300 mg daily for 14 or 21 days) plus conventional medicine showed no significant side effects^30^. In the preclinical study, we compared the acute toxicity of TBN (LD_50_: 858 mg/kg in mice by *i.v.*) and TMP (LD_50_: 351 mg/kg in mice by *i.v.*), TBN was two times less toxic than TMP. In addition, because TMP has activities of inhibiting platelet aggregation and lysing blood clots, we concern if TBN causes hemorrhage when the drug will presumably be used in conjunction with thrombolytic, such as tPA, and anti-platelet agent, such as aspirin. In the preclinical study, we evaluated the effect of TBN on anti-platelet aggregation, and demonstrated that TBN had marginal effect on platelet aggregation and there was no synergism with aspirin (data not shown). Based on the safety of TMP in clinical use and the preclinical safety evaluation of TBN, we speculate that TBN would be safe and has few side effects in human.

Here we used iTRAQ-based quantitative proteomic analyses to elucidate the molecular events involved in the long-term benefit effect of TBN. The major cellular processes observed in our proteomic changes were associated with the chronic phase of stroke, including angiogenesis, neurogenesis, astrocytosis and inflammation, revealing that TBN possibly regulates the brain tissue repair and recovery from stroke injury. In addition, several proteins related to various aspects of cellular energy metabolism and mitochondria were downregulated after TBN treatment. This suggests that one of the mechanisms involved in neuroprotection of TBN may be similar to one involved in hypothermia and ischemic preconditioning, which have been shown to exhibit remarkable neuroprotection in laboratory studies and correlated with the downregulation of tissue and cellular metabolism^31^,^32^.

CNP is a highly expressed enzyme in the myelin sheath and is believed to play an essential role in axon myelination^24^, which is essential for the proper function of nervous system^33^. The decreased CNP expression implies neuronal damage and demyelination in stroke^24^,^26^. Demyelination, loss of myelin sheath around an axon, causes impaired sensation, movement, cognition, or other function aspects depending on which nerves are involved^34^. Remyelination in the peri-infarct area has been observed in the central nerve system after focal ischemia^35^. In both proteomic analysis and Western blotting validation, TBN significantly upregulated the decreased CNP expression level in the stroke peri-infarct tissue. Thus, TBN may promote the axon remyelination.

Neurogenesis is an important step in neural development, which plays a vital role in neuronal repair after damage during disease processes^36^,^37^,^38^. After stroke injury, the brain attempts to repair neural cell damage through initiation of neurogenesis. It has been reported that TMP could promote the proliferation and differentiation of neuronal precursor cells^39^,^40^, enhance migration toward the ischemic region in the MCAo rat model^41^. Adult brain has the capacity for self-repair after stroke insults *via* neuronal replacement from endogenous precursors^42^ or a latent neurogenic program in the striatal astrocytes^43^. Our recent unpublished data suggest that TBN notably promotes polarization of cortical neurons *in vitro*, and increases the production of newly born (BrdU-positive) neurons in the peri-infarct area of rat MCAo models.

In the present study, all animal experiments were randomized and blinded by an independent researcher. Researchers remained blinded throughout behavioural, imaging, histological assessments. Groups were decoded at the end of each experiment upon statistical analysis. The design of this study imitated the clinical randomized and blinded trials to test the preclinical drug efficacy, which reduces the confounding effects of bias. Our approach may help to advance the translational roadblock, in which promising preclinical candidates fail to be translated to clinically effective treatments^44^,^45^. Recently, the clinical trial of TBN for ischemic stroke therapy has been approved by the China Food and Drug Administration.

In conclusion, TBN reduced cerebral infarction and improved neurological functions with unique multifunctional mechanisms of action in an ischemic stroke model of non-human primates. Finally, the therapeutic efficacy of neuroprotective TBN for ischemic stroke in preclinical findings should be further tested in clinical trials.

## Methods

### Monkey transient middle cerebral artery (MCA) occlusion (t-MCAo) model

Thirty adult male *Cynomolgus* macaques were used. Animals were 4–5 years of age and 3.0–4.0 kg of body weight at the start of the experiment. The animals were housed individually in stainless steel cages with 63 cm wide by 76 cm high by 76 cm deep (confirmation to NIH requirement) and maintained on a 12 h light-dark cycle (light on 8:00 to 20:00), had good visual and auditory interaction with other monkeys. All animals received water *ad libitum* and were provided monkey chow, supplemented with milk, fruit and seeds throughout the day. Primate-specific toys and audiovisual media were provided during light hours. Animals were fasted for 12 h before administration of anesthesia or behavioral testing.

The details of stroke surgical procedure were described in Supplementary Method. Two monkeys with failure of ischemic and/or reperfusion were excluded from further experiments. Three monkeys died within 3 h post-surgery and before saline or drug administration as a result of surgical/anesthetic complications. No animals died because of ischemic stroke or drug treatment during the experimental period, and no animals were excluded from data analysis at the end of the experiment.

Body weights were monitored and not significantly affected by surgery and drug treatment during the experimental period (see Supplementary Fig. S3B). The monkeys’ behaviour was measured for 28 days. Brain infarct size was inspected using magnetic resonance imaging (MRI) on days 7 and 28 after surgery. The representative images of *Tof-3D-multi-slab* scan at 7 days post stroke showed that the M1 segment of the left middle cerebral artery in all monkeys were open (see Supplementary Fig. S4), suggesting that surgical process of MCA reperfusion succeeded, no artery injury and reperfusion good.

Surgery, behavioural testing, MRI inspection and histological analysis were all done blinded to those who performed the experiments.

All procedures were performed in accordance with testing facility Standards of Practice (SOP) and regulations of Kunming Biomedical International (KBI Ltd., Yunnan Province, China). Animal experimental protocols were approved by the institutional animal care and use committee (IACUC) of KBI Ltd., which ensured humane and proper care of research animals. All efforts were made to minimize the numbers of animals used and ensure minimal suffering.

### Experimental groups

Thirty monkeys with successful *t*-MCAo surgery were block-randomly divided into five groups with 6 per group: (1) Model, stroke animals treated with saline; (2) 10 mg kg^−1^ TBN; (3) 30 mg kg^−1^; (4) 90 mg kg^−1^ TBN; (5) 90 mg kg^−1^ TBN delayed treatment. On the day of surgery, saline or drugs (dissolved in saline) were given (*i.v.* bolus, 1 mL kg^−1^) at 3 and 6 h (groups 1–4) or at 6 and 9 h (group 5) after *t*-MCAo. Drugs were then given twice daily for a total of 7 days post-surgery. TBN (Lot. No. 20101102, purity 99.3%) used in this study was synthesized by Shanghai Medicilon Inc. (Shanghai, China).

### Assessment of neurological behavior

The neurological severity score (NSS), based on that described by Kito *et al.*^46^, included assessments of consciousness, sensory and motor system function, and skeletal muscle coordination (see Supplementary Table 3). In addition to the NSS, we also tested the functional recovery of the contralateral limb using food catching. The monkeys were measured on their ability to freely catch food (apple pieces of 1 cm^3^ cubic shape). Before the stroke surgery, all monkeys were trained to successfully catch 15 pieces of apple within 5 min using their both limbs. When food catching task was performed, monkey was restrained in a special monkey chair (as shown in Supplementary Movie 1 and 2), their left limbs could only reach the left foot container, and their right limbs could only reach the right foot container. Then, the numbers of successful catching and total catching (times of touching the food container, including successful and failed catching) using both limbs were recorded. The success rate of food catching was calculated as: times of successful catching/times of total catching×100%.

### Evaluation of brain infarct size by MRI

For the imaging study, after the animals had been anesthetized by intramuscular administration of ketamine hydrochloride (10 mg kg^−1^) containing atropine sulfate (0.05 mg kg^−1^), MRI imaging was performed from the frontal to the posterior brain regions employing an MRI scanner (Siemens-MR-Verio 3.0, Siemens, Germany) with *Tof-3D-multi-slab*, *T1-f12d-tra* and *T2-tse-tra* scan. The sequence of *Tof-3D-multi-slab* was the cerebrovascular dynamic scanning for determining cerebral blood vessel integrity and patency. The sequence of *T1-f12d-tra* scan determined the gross anatomy map of animal brain tissue. The sequence of *T2-tse-tra* scan detected the infarct area of animal brain tissue, in which the infarct area showed high signal and normal brain tissue showed low signal. Transverse images with a 4-mm slice thickness were obtained from each *T2*-MRI sequence. The infarct size on *T2*-MRI imaging of the ischemic side was defined as the white portions and was compared to those on the contralateral side using Adobe Photoshop 7.0 software (San Jose, CA, USA).

### Tissue preparation

At termination of the experiment, monkeys were euthanized with a sodium pentobarbital overdose (150 mg kg^−1^, *i.v.*). The brain was removed and 4 mm brain slices (total 12 pieces from each brain) were sectioned coronally on ice with the brain immersed in cold saline (0.9%) for less than 15 min. For every brain slice within the areas of visible infarct, the normal cerebral cortex of the contralateral cerebral hemisphere and the peri-infarct area (adjacent to infarcted tissue) of the cerebral cortex from the ipsilateral hemisphere were dissociated for protein extraction and proteomics analysis. The tissues were immediately frozen in liquid nitrogen, and stored at −80 °C. The other brain slices were immersed in 4% paraformaldehyde to fix for 48 h. The tissues were then processed for histopathologic evaluation.

### Pharmacokinetics

Six *Cynomolgus* macaque monkeys were used for pharmacokinetic study. TBN were given intravenously at doses of 30 and 90 mg kg^−1^, 3 monkeys for each dose. Blood samples were collected through the hind limb vein using 5 mL heparinized syringes at 0, 2.5, 5, 10, 20, 40, 80 and 160 min post drug administration. Blood samples were centrifuged at 3,200 g for 10 min at 4 °C. Following centrifugation, plasma was collected and frozen until further analysis.

After the animals were anesthetized by intramuscular administration of ketamine hydrochloride (10 mg kg^−1^) containing atropine sulfate (0.05 mg kg^−1^), cerebrospinal fluid samples were collected from the cisterna magna at 15, 45, 60 and 120 min post drug administration. Drug concentrations were determined by HPLC analysis.

### Primary cortical neurons culture and neuroprotective assays

We prepared cortical cultures from E18 rat embryos (Experimental Animal Center of Sun Yat-Sen University) as previously described^47^. Cortical neurons were grown at 37 °C in a humidified incubator with 5% CO_2_/95% air atmosphere. After 4–6 h, unattached cells and debris were removed by replacing the initial medium with fresh neurobasal medium containing B27 supplements. Subsequent partial medium replacement was carried out twice a week and cultures at 11 days were used for the experiments. TBN at indicated concentrations were applied 2 h before exposure to glutamate (200 μM), and the cells were cultured for another 24 h. Cell viability was assessed by 3-(4,5-dimethylthiazol-2-yl)-2, 5-diphenyltetrazolium bromide (MTT) assay^15^.

The neuroprotective effect of TBN was further assessed by attenuation of LDH release on OGD-insulted cortical neurons. TBN at indicated concentrations were applied 2 h before exposure to OGD for 4 h, and the cells were cultured for another 24 h. LDH release assay was conducted according to our previous report^48^.

### Isobaric tags for relative and absolute quantitation (iTRAQ)-based quantitative proteomic analyses

The details of iTRAQ-based quantitative proteomic analyses were described in Supplementary Methods, including proteomics sample preparation, iTRAQ labelling, liquid chromatography, mass spectrometry and data analysis, and iTRAQ ratio calculations and criteria for the selection of dysregulated proteins.

### Western blotting

For protein extraction, tissues were extracted with RIPA lysis buffer containing 1% PMSF and 1% protease inhibitor cocktail on ice for 10 min. The lysates were centrifuged (12,500 × *g*, 20 min, 4 °C) and the supernatants were then collected and stored at –80 °C until use. Protein contents were assayed using a BCA protein quantification kit (Pierce, Rockford, IL, USA). Protein samples (30 μg) were resolved using SDS-PAGE and transferred to PVDF membranes. The immunoblots were analyzed with the appropriate primary antibodies (1:1000). Horseradish peroxidase-conjugated secondary antibodies (1:2500) were used to detect the proteins of interest through enhanced chemiluminescence.

### Immunohistochemistry

The fixed brain slices were dehydrated with graded alcohol, embedded in paraffin wax, and coronally sectioned at 6 μM. Immunohistochemistry of sections was performed using antibodies against glial fibrillary acidic protein (GFAP, Cat.no. MAB360, Millipore) and neuronal nuclei (NeuN, Cat.no. MAB 377, Millipore) to evaluate the infiltration of inflammatory cells and viability of neurons. The positive cells were randomly counted in 5 areas per section and 3 sections of each monkey from 6 monkeys per group, and then averaged the data.

### Data and statistical analyses

We carried out statistical analysis with Prism 6.0 software (GraphPad Software). Unless otherwise specified, all data were expressed as mean ± s.e.m, and multiple comparisons were assessed by one-way ANOVA followed by the Tukey’s *post hoc* test for significance. The significance level was set as *P* < 0.05.

## Additional Information

**How to cite this article**: Zhang, Z. *et al.* Tetramethylpyrazine nitrone, a multifunctional neuroprotective agent for ischemic stroke therapy. *Sci. Rep.* **6**, 37148; doi: 10.1038/srep37148 (2016).

**Publisher’s note**: Springer Nature remains neutral with regard to jurisdictional claims in published maps and institutional affiliations.

## Supplementary Material

Supplementary Information

Supplementary Dataset 1

Supplementary Dataset 2

Supplementary Dataset 3

Supplementary Movie 1

Supplementary Movie 2

## Figures and Tables

**Table 1:** Neurological deficit scores of stroke monkeys at 1 and 4 weeks post-surgery.

	1 week							4 weeks						
	Total neurological deficit (0–77)	Consciousness (0–29)	Sensory system (0–12)	Motor system (0–17)	Skeletal muscle coordination (0-19)	Successful times of right hand catching (0–15)	Total times of right hand catching (0–15)	Total neurological deficit (0–77)	Consciousness (0–29)	Sensory system (0–12)	Motor system (0–17)	Skeletal muscle coordination (0-19)	Successful times of right hand catching (0–15)	Total times of right hand catching (0–15)
Model	21.7 ± 4.1	5.7 ± 0.8	2.0 ± 0.6	7.0 ± 1.7	7.0 ± 1.3	1.0 ± 1.0	3.5 ± 1.0	15.5 ± 1.7	4.3 ± 0.3	2.0 ± 1.0	4.3 ± 0.3	5.0 ± 0.4	1.8 ± 1.6	4.5 ± 0.8
10 mg kg^−1^ @3 h	20.7 ± 5.6	5.0 ± 0.7	3.3 ± 1.8	6.0 ± 2.0	6.3 ± 1.5	7.5 ± 2.6	8.0 ± 2.4	11.5 ± 2.3	2.7 ± 0.8*	1.5 ± 1.0	3.0 ± 0.7	4.0 ± 0	7.5 ± 3.4*	10.0 ± 2.5*
30 mg kg^−1^ @3 h	18.7 ± 3.4	5.3 ± 0.7	2 ± 0.6	5.7 ± 1.7	5.7 ± 1.0	8.8 ± 2.6*	10.7 ± 2.6*	14.0 ± 1.6	4.3 ± 0.3	1.0 ± 0.6	4.0 ± 0.5	4.7 ± 0.4	7.7 ± 2.4*	9.0 ± 2.4*
90 mg kg^−1^ @3 h	19.3 ± 2.6	5.3 ± 0.7	3 ± 0.8	5.0 ± 1.0	6.0 ± 0.9	3.3 ± 1.8	7.8 ± 3.2	11.3 ± 2.8	3.0 ± 1.0	0.5 ± 0.5	3.5 ± 1.0	4.3 ± 1.0	6.2 ± 2.9	7.0 ± 3.0
90 mg kg^−1^ @6 h	17.3 ± 1.5	5.0 ± 0.4	2.5 ± 0.5	5.0 ± 0.6	5.3 ± 0.4	6.3 ± 2.3	11.7 ± 2.3*	14.0 ± 0.6	4.0 ± 0	2 ± 0.6	4.0 ±0	4.0 ± 0	9.8 ± 2.4*	11.5 ± 1.9*

*P < 0.05 versus Model. Data at 1 and 4 weeks were separately analyzed by one-way ANOVA, followed by Tukey’s *post hoc* test. ‘@3 h’ and ‘@6 h’ meant TBN treatment initiating at 3 h and 6 h post-ischemia.
