# Supplementary material for: Tetramethylpyrazine nitrone, a multifunctional neuroprotective agent for ischemic stroke therapy
Source: Sci Rep. 2016 Nov 14;6:37148. doi: 10.1038/srep37148 (PMC5107909; doi:10.1038/srep37148)
Supplement: Supplementary Information [file srep37148-s1.pdf]

Supplementary information

Tetramethylpyrazine nitrone, a multifunctional neuroprotective agent for ischemic stroke therapy

Zaijun Zhang [1, †], Gaoxiao Zhang [1, †], Yewei Sun [1, †], Samuel S. W. Szeto [2], Henry C. H. Law [2], Quan Quan [2], Guohui Li [2], Pei Yu [1], Eiketsu Sho [3], Michael K. W. Siu [4], Simon M.Y. Lee [5], Ivan K. Chu [2, *], Yuqiang Wang [1, *]

[1] Institute of New Drug Research and Guangzhou Key Laboratory of Innovative Chemical Drug Research in Cardio-cerebrovascular Diseases, Jinan University College of Pharmacy, Guangzhou, 510632, China.

[2] Department of Chemistry, the University of Hong Kong, Hong Kong, China.

[3] Kunming Biomed International and National Engineering Research Center of Biomedicine and Animal Science, Kunming, 650500, China.

[4] Department of Chemistry and Biochemistry, University of Windsor, Windsor, Ontario, Canada.

[5] State Key Laboratory of Quality Research in Chinese Medicine, Institute of Chinese Medical Sciences, University of Macau, Macau, China.

†These authors contributed equally to this work.

* **Corresponding authors**: Yuqiang Wang, Institute of New Drug Research and Guangzhou Key Laboratory of Innovative Chemical Drug Research in Cardio-cerebrovascular Diseases, Jinan University College of Pharmacy, Guangzhou, 510632, China. Email: yuqiangwang2001@yahoo.com; Ivan K. Chu, Department of Chemistry, the University of Hong Kong, Hong Kong, China. Email: ivankchu@hku.hk.

This Supplementary formation document includes Supplementary methods, Supplementary Figs. S1-S4, Supplementary Tables S1- S3, Supplementary Dataset 1- 3 and Supplementary Movie 1-2. Please note Supplementary Dataset 1- 3 and Supplementary Movie 1-2 are uploaded as separate Excel and SWF documents.

Supplementary Fig. S1. Experimental setup for proteomic study.

Supplementary Fig. S2. Immunoblotting validation of dysregulated proteins identified by proteomics analysis.

Supplementary Fig. S3. Relative cerebral blood flow (*r*CBF) and body weight changes of *t*-MCAO monkeys.

Supplementary Fig. S4. Representative 2D and 3D cerebrovascular structures by *Tof-3D-multi-slab* MRI scan.

Supplementary Table S1. Dysregulated proteins.

Supplementary Table S2. Dysregulated proteins not classified to cellular processes associated with the chronic phase of stroke in the Figure 3.

Supplementary Table S3. Neurological deficit score for monkey.

Supplementary Dataset 1. Identified proteins list.

Supplementary Dataset 2. Quantified proteins list.

Supplementary Dataset 3. Dysregulated proteins list.

Supplementary Movie 1. Stroke monkey without treatment. Ischemic stroke dramatically destroyed ability of the monkey contralateral, stroke-affected arm to acquire food.

Supplementary Movie 2. Stroke monkey with 30 mg kg^{-1} TBN treatment. TBN (30 mg kg^{-1}) treatment obviously restored ability of the monkey contralateral, stroke-affected arm to acquire food.

Supplementary Methods

Stroke model

Before surgery, monkeys were trained and tested on a number of neurological tests, including habits and behavior observing and recording beside cages under consciousness; tractive walk and the moving paces observing and recording; balance beam test; upper limb food taking. The trainings were implemented three times each week for at least 3 weeks.

Monkey anaesthesia was induced by intramuscular injection of ketamine (10 mg kg^{-1}) right after intramuscular injection of atropine sulfate (0.05 mg kg^{-1}). Anaesthesia was maintained with isoflurane inhalation (0.5-2%) mixed in oxygen during the entire surgery. After turning a large cranial flap and opening the dura over the left lateral frontal cortex, the left M1 segment of the MCA, 2 mm medial to the olfactory tract, was transiently occluded by a 5 mm titanium artery clip. Artery clamp was removed after 4 h of MCAo to restore MCA blood flow. Relative cerebral blood flow (*r*CBF) was measured on the area of frontal cortex using a laser Doppler blood flow meter (PeriMed). Only monkeys with a relative cerebral blood flow decrease below 65% of baseline upon occlusion and recovery up to 65% of baseline upon reperfusion were deemed to undergo successful MCA occlusion and reperfusion (see Supplementary Fig. S3A online). Two monkeys with failure of ischemic and/or reperfusion was excluded from further experimentation. Three monkeys died within 3 h post-surgery and before saline or drug administration as a result of surgical/anesthetic complications. Post-surgically, monkeys were placed in the recovery room on a heating pad, and were closely monitored for the following 48 h. Fluids of 0.9% NaCl with 5% glucose were intravenous given to avoid hypoglycemia. Buprenex was administrated for analgesia. Cefazolin (25 mg kg^{-1}) was also given by i.m. twice daily for total 3 days. No animals died because of ischemic stroke or drug treatment during the experimental period, and no animals were excluded from data analysis at the end of experiment.

ITRAQ-based quantitative proteomic analyses

Proteomics sample preparation and isobaric tags for relative and absolute quantitation (iTRAQ) labelling

The brain tissue used for proteomic study was from animals treated with saline and 30 mg/kg TBN initiating at 3 and 6 h following ischemia, and brain tissues were taken at 4 weeks

following stroke. To extract the protein, the frozen brain tissues were first rinsed with ice-cold RIPA buffer containing 1x phosphatase and protease inhibitors and minced into small pieces. RIPA buffer was then added to the minced tissue and homogenized with a Tissue Tearor (Biospec, Bartlesville, OK) at full speed on ice for approximately 10 to 30 s. The samples were vortexed briefly, ultrasonicated for 10 cycles (10 s ON/10 s OFF) at 4°C, then centrifuged at 15,000 rpm for 15 min at 4°C. The extracted proteins, solubilized in the supernatant, were purified with acetone precipitation and overnight incubation at -20°C. The precipitated proteins were collected by centrifugation at 4000 rpm for 15 min at room temperature, re-dissolved in 8 M urea and quantified using Bradford assay kit. On filter protein digestion was carried out in the same manner as previously described in Quan *et al.* (*1*). The tagging strategy used for the samples is illustrated in (Fig. S2). The samples were lyophilized and stored at –80 ℃ until required for use.

Liquid chromatography

The iTRAQ samples were reconstituted in 0.5% formic acid and analyzed with online multidimensional liquid chromatography platforms. These included two-dimensional RP-SCX-RP (*2*) and PGC-RP (*3*) platforms that were installed on an Eksigent nanoLC Ultra 2D Plus (AB SCIEX, Framingham, MA). Other platforms, such as concatenation RP-RP (*4*), three-dimensional HILIC-SCX-RP (*5*) and four-dimensional RP-SA(C)X-RP (*1*), were assembled with an Agilent 1200 series nano pump, capillary pumps and a 10 well-plate auto-sampler (Agilent Technologies, Wilmington, DE). Sample injections and flow channel switching were conducted using Valco valves. The detailed assembly of the valve systems, specifications of columns and platform operation conditions can be found in the respective references.

MS and data analysis

The MS experiments were carried out using a TripleTOF 5600 system fitted with a Nanospray III source (AB SCIEX, Concord, ON, Canada) with the following applied parameters: ion spray voltage, 2.5 kV; curtain gas, 30 psi; declustering potential, 100 V; curtain gas, 30 psi; nebulizer gas, 15 psi; interface heater temperature, 150 ^{o}C. For information-dependent acquisition, full scans were acquired over 250 ms in the range of *m/z* 400-1250 over 250 ms. The 15 most abundant peaks that exceeded 125 counts and carried charge state +2 to +5 were selected for subsequent MS/MS acquisition over 150 ms in the range of *m/z* 100-1500. The iTRAQ rolling collision energy option was enabled from the controlling software interface with the dynamic

exclusion time of the acquired ions set at 20 s. The acquired data were searched against the theoretical spectra generated from the protein sequences in the NCBI *Macaca fascicularis* database (obtained in January 2014 with 56,572 entries; http://www.ncbi.nlm.nih.gov/protein) using the Paragon algorithm (*6*) in ProteinPilot 4.5 (Applied Biosystems, Framingham, MA, USA). In all searches, trypsin was set as the enzyme used and IAA was chosen as the cysteine alkylation reagent. The mass tolerance used in the search was predetermined in the software with the instrument option set as TripleTOF 5600. The search effort and the ID focus options in the software were set as "thorough" and "biological modifications", respectively. The plug-in Proteomics System Performance Evaluation Pipeline (PSPEP) featured in ProteinPilot 4.5, was employed for analysis of the false discovery rate (FDR)(*7*). Protein groups with local FDR <5% were considered as identified proteins (*8, 9*).

iTRAQ ratio calculations and criteria for the selection of dysregulated proteins

In addition to the criteria imposed for protein identification, quantification also required the presence of at least 4 spectra possessing $\geq$ 95% confidence. The protein expression ratios were calculated using the weighted average of the natural logarithms of the observed peptide ratios, as described in ProteinPilot manual. Proteins were considered to be dysregulated (up- and/or downregulated) if their expression ratios were $\geq$ 1.23 or $\leq$ 0.81 and possessed *p*-values $\leq$ 0.05. The *p*-values were determined using a one-tailed *t*-test on the iTRAQ reporter ion ratios calculated from the observed spectra. For a protein to be categorized as dysregulated in either the stroke or TBN treatment conditions, the protein expression ratios from both biological replicates had to exhibit a consistent trend and pass the aforementioned criteria.

References

1. Q. Quan, S. S. Szeto, H. C. Law, Z. Zhang, Y. Wang, I. K. Chu, Fully Automated Multidimensional Reversed-Phase Liquid Chromatography with Tandem Anion/Cation Exchange Columns for Simultaneous Global Endogenous Tyrosine Nitration Detection, Integral Membrane Protein Characterization, and Quantitative Proteomics Mapping in Cerebral Infarcts. *Anal Chem* **87**, 10015-10024 (2015).
2. H. C. Law, R. P. Kong, S. S. Szeto, Y. Zhao, Z. Zhang, Y. Wang, G. Li, Q. Quan, S. M. Lee, H. C. Lam, I. K. Chu, A versatile reversed phase-strong cation exchange-reversed phase (RP-SCX-RP) multidimensional liquid chromatography platform for qualitative and quantitative shotgun proteomics. *Analyst* **140**, 1237-1252 (2015).
3. Y. Zhao, S. S. Szeto, R. P. Kong, C. H. Law, G. Li, Q. Quan, Z. Zhang, Y. Wang, I. K. Chu, Online two-dimensional porous graphitic carbon/reversed phase liquid

chromatography platform applied to shotgun proteomics and glycoproteomics. *Anal Chem* **86**, 12172-12179 (2014).

4. Y. Wang, F. Yang, M. A. Gritsenko, T. Clauss, T. Liu, Y. Shen, M. E. Monroe, D. Lopez-Ferrer, T. Reno, R. J. Moore, R. L. Klemke, D. G. Camp, 2nd, R. D. Smith, Reversed-phase chromatography with multiple fraction concatenation strategy for proteome profiling of human MCF10A cells. *Proteomics* **11**, 2019-2026 (2011).

5. Y. Zhao, H. C. Law, Z. Zhang, H. C. Lam, Q. Quan, G. Li, I. K. Chu, Online coupling of hydrophilic interaction/strong cation exchange/reversed-phase liquid chromatography with porous graphitic carbon liquid chromatography for simultaneous proteomics and N-glycomics analysis. *J Chromatogr A* **1415**, 57-66 (2015).

6. I. V. Shilov, S. L. Seymour, A. A. Patel, A. Loboda, W. H. Tang, S. P. Keating, C. L. Hunter, L. M. Nuwaysir, D. A. Schaeffer, The paragon algorithm, a next generation search engine that uses sequence temperature values and feature probabilities to identify peptides from tandem mass spectra. *Mol. Cell. Proteomics* **6**, 1638-1655 (2007).

7. W. H. Tang, I. V. Shilov, S. L. Seymour, Nonlinear fitting method for determining local false discovery rates from decoy database searches. *J Proteome Res* **7**, 3661-3667 (2008).

8. P. Jagtap, T. McGowan, S. Bandhakavi, Z. J. Tu, S. Seymour, T. J. Griffin, J. D. Rudney, Deep metaproteomic analysis of human salivary supernatant. *Proteomics* **12**, 992-1001 (2012).

9. A. Mange, A. Goux, S. Badiou, L. Patrier, B. Canaud, T. Maudelonde, J. P. Cristol, J. Solassol, HDL proteome in hemodialysis patients: a quantitative nanoflow liquid chromatography-tandem mass spectrometry approach. *PLoS One* **7**, e34107 (2012).

Supplementary Fig. S1

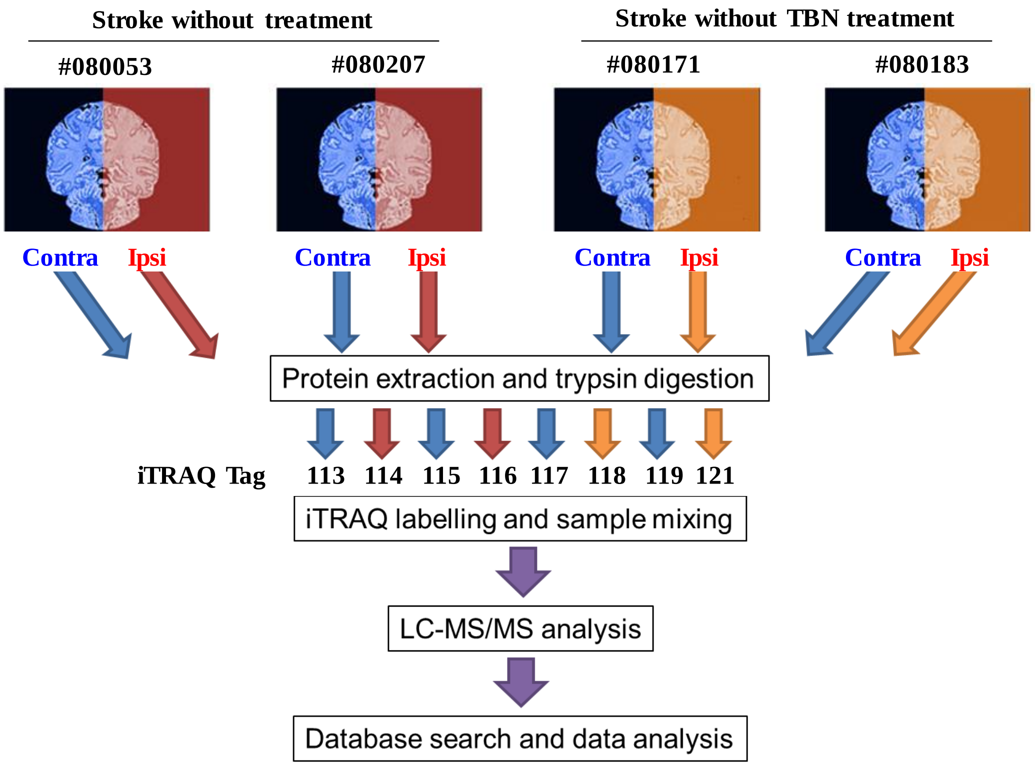

Supplementary Fig. S1. Experimental setup for proteomic study. The experimental condition, *M. fascicularis* identifier (#080053, #080207, #080171 and #080183), and sample location with respect to the side of the cerebral cortex from which the tissue was taken, are indicated for each of the iTRAQ tags used in the experiment. 'Contra' and 'Ipsi' refer to the contralateral and ipsilateral sides of the cerebral cortex, respectively, in relation to the site of the *t*-MCAo surgery.

Supplementary Fig. S2

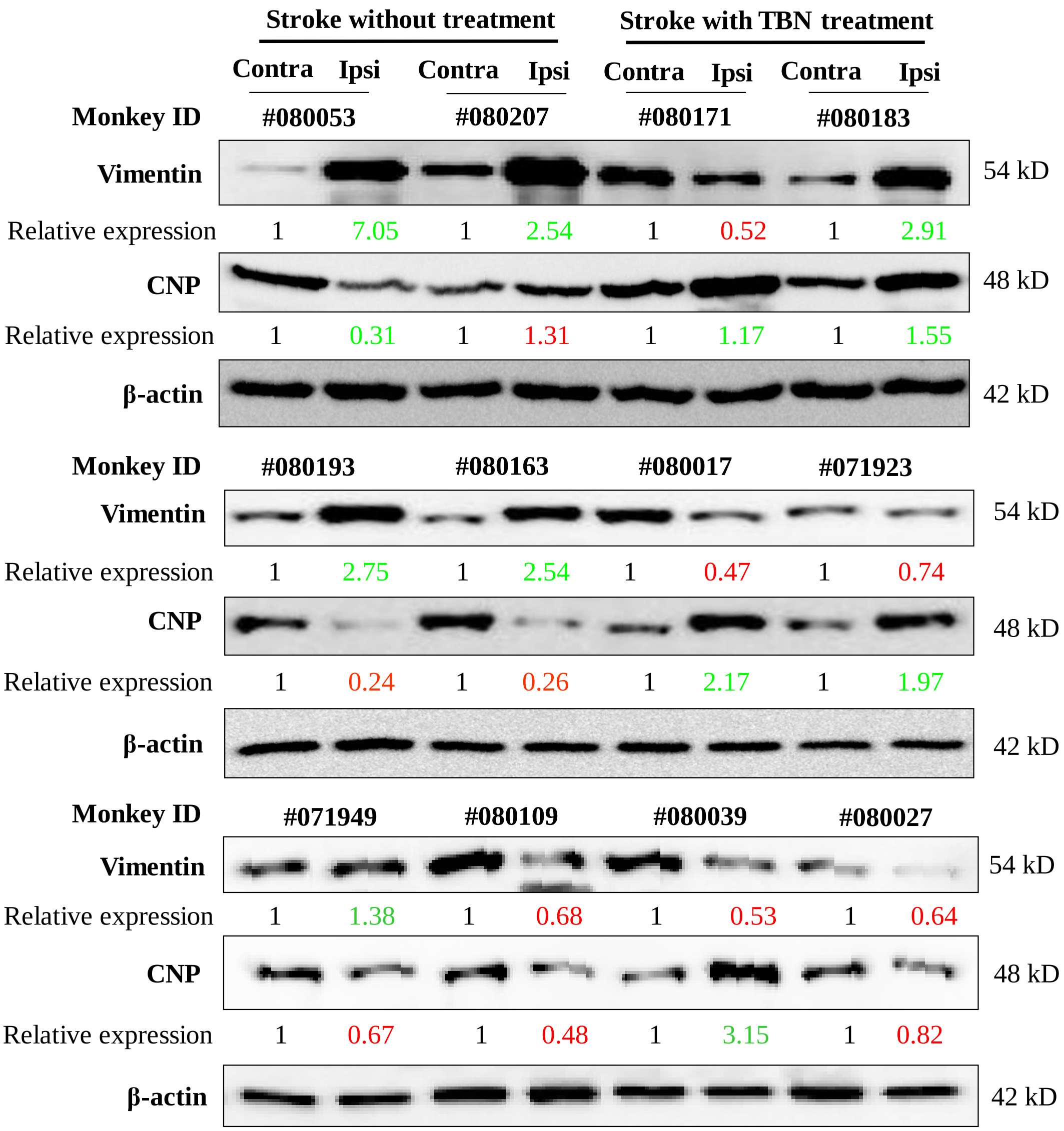

Supplementary Fig. S2. Immunoblotting validation of dysregulated proteins identified by proteomics analysis.

Supplementary Fig. S3

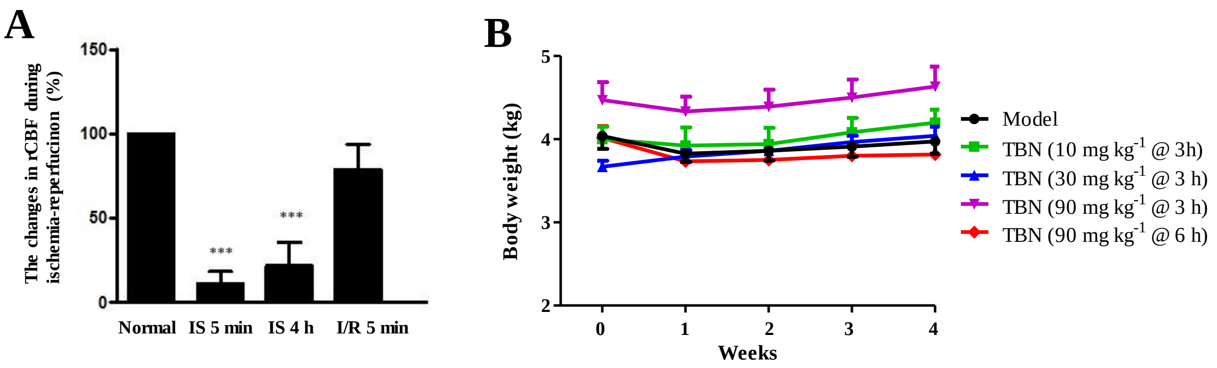

Supplementary Figure 3. Relative cerebral blood flow (*r*CBF) and body weight changes of *t*-MCAO monkeys. (**A**) *r*CBF change during MCAo surgery. (**B**) Body weight change. All data are mean ± s.e.m. n=34 monkeys in **a**; ****P*<0.001 versus normal group (before MCAo operation); One-way ANOVA and Turkey's test. 'IS 5 min' and 'IS 4 h' are 5 min and 4 h post-ischemia; 'I/R 5 min' is 5 min post-ischemia/reperfusion. In **b**, n=6 monkeys for Model and all TBN treatment groups.

Supplementary Fig. S4

2D cerebrovascular structure   3D cerebrovascular structure

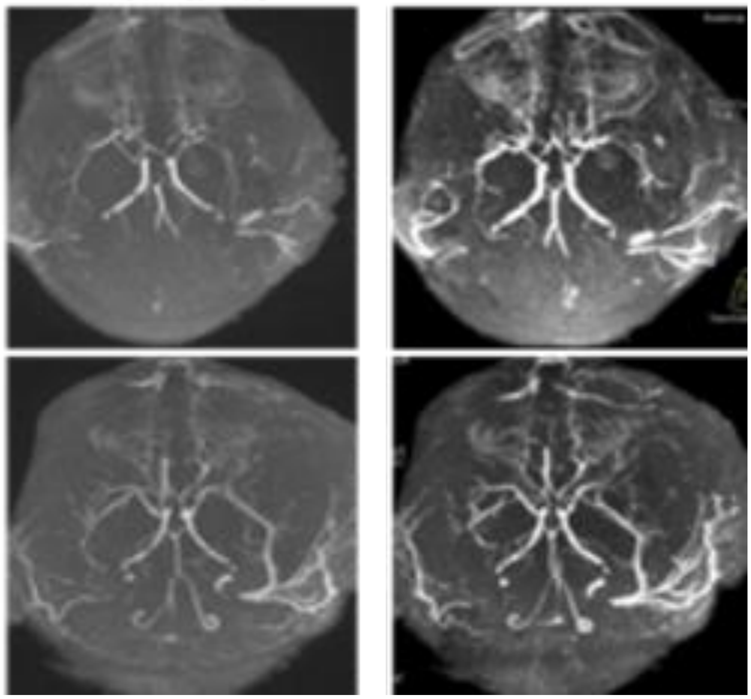

Supplementary Fig. S4. Representative 2D and 3D cerebrovascular structures by *Tof-3D-multi-slab* MRI scan.

Supplementary Table S1. Dysregulated proteins.

GI number	Protein name	Stroke	TBN 30 mg kg^{-1} @ 3 h
544512024	Apolipoprotein E [a, b, c, d]	↑	-
544486489	CD44 molecule (Indian blood group) [a, b, c]	↑	-
544521119	Proprotein convertase subtilisin/kexin type 1 inhibitor [b]	↑	-
544402345	S100 calcium binding protein A6	↑	-
544440333	Calcium/calmodulin-dependent protein kinase II alpha [b]	↓	-
544422137	Lactate dehydrogenase B	↓	-
544526346	Tubulin, beta 4A class IVa [c]	↓	-
544500058	2',3'-cyclic nucleotide 3' phosphodiesterase [b]	-	↑
544412649	Claudin 11	-	↑
544489680	Crystallin, alpha B [a]	-	↑
544523796	Filamin A, alpha [a, c]	-	↑
544470012	Keratin 1, type II [a, c]	-	↑
544499934	Keratin 10, type I [c]	-	↑
544461954	Lectin, galactoside-binding, soluble, 1 [a, b, c]	-	↑
544520248	Monoamine oxidase B [c, d]	-	↑
544505129	Myelin basic protein [b, c]	-	↑
544429419	Myelin oligodendrocyte glycoprotein [b, c]	-	↑
544413445	Myelin-associated oligodendrocyte basic protein	-	↑
544463659	Neurofilament, heavy polypeptide [b]	-	↑
544450971	Neurofilament, light polypeptide [b]	-	↑
544522519	Plastin 3	-	↑
544432802	Albumin [c]	-	↓
544418443	ATP synthase, H^+ transporting, mitochondrial F_o complex, subunit F6	-	↓
544407834	ATPase inhibitory factor 1 [a]	-	↓
544436461	Brain abundant, membrane attached signal protein 1 [b]	-	↓
544449011	Calmodulin [b]	-	↓
544496553	Complement component 1, Q subcomponent binding protein [c]	-	↓
544442918	Creatine kinase U-type, mitochondrial [b, c]	-	↓
544449659	Creatine kinase, brain [b, c]	-	↓
544444615	Cytochrome c oxidase subunit Va	-	↓
544479280	Cytochrome c oxidase subunit Vb	-	↓

544406046	Cytochrome c, somatic [a]	-	↓
544422188	Dihydrolipoamide dehydrogenase	-	↓
544459307	Glutamic-oxaloacetic transaminase 1, soluble	-	↓
544517535	Glutamic-oxaloacetic transaminase 2, mitochondrial	-	↓
544419424	Malate dehydrogenase 2, NAD (mitochondrial)	-	↓
544516099	NADH dehydrogenase (ubiquinone) 1, alpha/beta subcomplex, 1, 8 kDa	-	↓
544509062	Peroxiredoxin 2	-	↓
544459173	Phosphoglycerate mutase 1 (brain)	-	↓
544516342	Proline-rich transmembrane protein 2	-	↓
544457999	Prosaposin [a, b, c, d]	-	↓
544417864	Purkinje cell protein 4	-	↓
544402317	S100 calcium binding protein A1	-	↓
544418295	Superoxide dismutase 1, soluble [a, b, c, d]	-	↓
544494926	Annexin A1 [c]	↑	↑
544453107	Fatty acid binding protein 5 (psoriasis-associated) [c]	↑	↑
544500577	Glial fibrillary acidic protein [b, c]	↑	↑
544492641	Tenascin C [a, b, c]	↑	↑
544456116	Vimentin [a, b, c]	↑	↑
544424506	Superoxide dismutase 2, mitochondrial [b, c]	↑	↓
544522169	Proteolipid protein 1 [b, c, d]	↓	↑

[#] Inclusion in this list required that the iTRAQ protein ratios of both biological replicates within the same group (either stroke or TBN 30 mg/mg @ 3h) display a consistent trend of above or below the prescribed cutoff values (>1.23 or <0.81) and be statistically significant (P<0.05). Overall upregulation was indicated by the symbol (↑) and labelled in red; overall downregulation was indicated by the symbol (↓) and labelled in green. Proteins exhibiting iTRAQ ratios that did not pass the cutoff values or inconclusive trends were labelled with the symbol (-).

[a] Proteins involved in angiogenesis

[b] Proteins involved in neurogenesis and synaptogenesis

[c] Proteins involved in inflammation

[d] Proteins involved in reactive astrocytosis

Supplementary Table S2. Dysregulated proteins not classified to cellular processes associated with the chronic phase of stroke in the Figure 3.

Gene symbol	Protein name	Stroke monkeys	TBN treatment monkeys	Remarks
S100A6	S100 calcium binding protein A6	↑	-	
LDHB	Lactate dehydrogenase B	↓	-	
CLDN11	Claudin 11	-	↑	
MOBP	Myelin-associated oligodendrocyte basic protein	-	↑	
PLS3	Plastin 3	-	↑	
ATP5J	ATP synthase, H+ transporting, mitochondrial Fo complex, subunit F6	-	↓	mitochondrial protein
COX5A	cytochrome c oxidase subunit Va	-	↓	mitochondrial protein
COX5B	cytochrome c oxidase subunit Vb	-	↓	mitochondrial protein
DLD	dihydrolipoamide dehydrogenase	-	↓	mitochondrial protein
GOT1	glutamic-oxaloacetic transaminase 1, soluble	-	↓	
GOT2	glutamic-oxaloacetic transaminase 2, mitochondrial	-	↓	mitochondrial protein
MDH2	Malate dehydrogenase 2, NAD (mitochondrial)	-	↓	mitochondrial protein
NDUFAB1	NADH dehydrogenase (ubiquinone) 1, Alpha/beta subcomplex, 1, 8kDa	-	↓	mitochondrial protein
PCP4	Purkinje cell protein 4	-	↓	
PGAM1	Phosphoglycerate mutase 1 (brain)	-	↓	
PRDX2	Peroxiredoxin 2	-	↓	
PRRT2	Proline-rich transmembrane protein 2	-	↓	
S100A1	S100 calcium binding protein A1	-	↓	

Red: Upregulated in both stroke monkeys;
Yellow: Upregulated in both treatment monkeys;
Green: Downregulated in both stroke monkeys;
Blue: Downregulated in both treatment monkeys

Supplementary Table S3. Neurological deficit score for monkey.

**Neurological deficit score**	
Category	Score
1) Consciousness	(range 0–29)
Normal, consistently alert	0
Conscious and aggressive	4
Conscious and evasive	6
Conscious but slow response	8
Drowsiness, aroused with stimulation	10
Lethargy, eyes opened by intense stimulation	16
Stupor, aroused with persistent stimulation	20
Light coma, reflex movement only	24
Deep coma, no movement	28
Death	29
2) Sensory system score (contralateral to ischemic side)	(range 0–12)
Facial sensation Reacts consistently to touch on any area of face	0
Absent, does not react to touch on any area of face	3
**Swallowing Reflex**	
Normal swallowing	0
Absent ,tongue deflect one side and dysphagia	3
**Blink reflex**	
Company with stimulation	0
Absent	3
**Pinna reflex**	
Response to ear twitch	0
Absent, no response to ear twitch	3
**Pain reflex**	
Strong, quick, complete withdrawal from toe pinch	0
Weak, slow, incomplete or inconsistent withdrawal from toe pinch	3
Absent, no withdraw from toe pinch	5
Death	12
3) Motor system score (contralateral to ischemic side)	(range 0–17)
**Upper limb (grasp and movement)**	
Normal	0
Reduced strength and disharmonious movement	2
Paralyzed and grasp inability	4

Lower limb (grasp and movement)	
Normal	0
Raise with flexion of knee	2
Can move but cannot raise	4
Paralyzed and cannot move	6
**Upper limb tone**	
Normal	0
Overtly spastic or flaccid	3
**Lower limb tone**	
Normal	0
Overly spastic or flaccid	3
Death	17
4) Skeletal muscle coordination	(range 0–19)
Normal, walks normally	0
Minimal incoordination, walks with some gait impairment	4
In coordinated, and cannot climb a perch	6
Stands independently, dysbasia	10
Sit; only able to circle with stimulation	12
Pose with lateral or dorsal recumbency	16
No movement	18
Death	19

Supplementary Dataset 1. Identified proteins list.

Supplementary Dataset 2. Quantified proteins list.

Supplementary Dataset 3. Dysregulated proteins list.

Supplementary Movie 1. Stroke monkey without treatment. Ischemic stroke dramatically destroyed ability of the monkey contralateral, stroke-affected arm to acquire food.

Supplementary Movie 2. Stroke monkey with 30 mg/kg TBN treatment. TBN (30 mg/kg) treatment obviously restored ability of the monkey contralateral, stroke-affected arm to acquire food.
